# Exploring providers’ perspectives of a community based TB approach in Southern Ethiopia: implication for community based approaches

**DOI:** 10.1186/s12913-015-1149-9

**Published:** 2015-11-09

**Authors:** Daniel G. Datiko, Mohammed A. Yassin, Olivia Tulloch, Girum Asnake, Tadesse Tesema, Habiba Jamal, Paulos Markos, Luis E. Cuevas, Sally Theobald

**Affiliations:** TB REACH Project, Sidama Zone, Hawassa, Ethiopia; Liverpool School of Tropical Medicine, Liverpool, L3 5QA UK; Global Fund to Fight AIDS, Tuberculosis and Malaria, Geneva, Switzerland; Department of International Public Health, Liverpool School of Tropical Medicine, Liverpool, L3 5QA UK; Liverpool School of Tropical Medicine, Pembroke Place, Liverpool, L3 5QA UK

## Abstract

**Background:**

There is increasing interest in the role of close-to-community providers in supporting universal health coverage, but questions remain about the best approaches to supporting and motivating these providers, and the optimal package they can deliver indifferent contexts and support required. We report on the experiences of different health providers involved in a community based intervention to support access to tuberculosis diagnosis and treatment in Southern Ethiopia.

**Methods:**

The aim of the study is to explore the experiences of health providers in delivering a community-based tuberculosis package in southern Ethiopia and to draw lessons for community-based programmes. A qualitative methodology was used. Methods included in-depth interviews (IDIs, n= 37) with all health provider groups: Community health promoters (CHPs), health extension workers (HEWs), district supervisors and laboratory technicians were undertaken to obtain a detailed understanding of the experiences of providers in the community based tuberculosis package. These were complemented with cadre specific focus group discussions (n= 3). We used the framework approach for qualitative analysis.

**Results:**

The key theme that emerged was the positive impact the community-based intervention had on vulnerable groups’ access to diagnosis, care and treatment for tuberculosis. Providers found the positive feedback from, and visible impact on, communities very motivating. Other themes related to motivation and performance included supervision and support; learning new skills; team problem solving/ addressing challenges and incentives. Against the backdrop of the Ethiopian Health Extension Programme (HEP), HEWs were successfully able to take on new tasks (collecting sputum and preparing smears) with additional training and appropriate support from supervisors, laboratory technicians and CHPs.

**Conclusion:**

All categories of providers were motivated by the high visible impact of the community-based intervention on poor and vulnerable communities and households. HEWs role in the community-based intervention was supported and facilitated through the structures and processes established within the community-based intervention and the broader nation-wide Health Extension Programme. Within community based approaches there is need to develop context embedded strategies to support, sustain and motivate this critical cadre who play a pivotal role in linking health systems and rural communities.

## Background

International and national decision-makers are once again turning to close-to-community providers, such as community health workers (CHWs), to strengthen health systems and as part of the push towards universal access and delivery of the Millennium Development Goals. Investments in CHW programmes followed the Alma Alta Declaration in 1970, but in many contexts the programmes went into decline due to lack of support and legitimacy, political instability, neo-liberal economic policies or difficulties in financing [1]. CHWs canbe considered as an umbrella term for a diverse range of close-to-community providers; they are increasingly employed in resource-limited settings where facility-based health services are inaccessible to the poorest and disadvantaged in society. Studies have illustrated the role that CHWs can have in supporting equity inthe delivery of health services to the vulnerable and the poor in marginalised communities [2, 3]. The importance of their interaction with other community level providers is also now becoming recognised [4]. Less is known however from the providers’ perspectives, such as their experience of human resources support for supervision or workload management [5], and the challenges or opportunities they face in delivering their work [6].

Tuberculosis (TB) control is one of many areas that CHWs have been recognized as making a valuable contribution. To date this contribution has primarily focused on direct observation of treatment [7]. Evaluations have concluded that involvement of CHWs in TB treatment support can substantially increase treatment completion rates as compared to facility-based services [8]. Studies have also demonstrated that community-based care, such as deploying CHWs in treatment support for TB, is more cost-effective than other forms of care [8]. The role of CHWs in supporting universal health coverage in TB case finding and diagnosis however is less well documented.

Ethiopia has substantially invested inclose-to-community services and providers: in 2003 the Ethiopian government introduced the Health Extension Program (HEP) to promote universal coverage of primary health care. Through the HEP a cadre of female Health Extension Workers (HEWs) who provide basic curative and preventive health services in every community has been deployed. Two HEWs are employed in each *kebele* (the smallest administrative unit), they are trained for one year and receive a salary; in common with other types of CHWs they are embedded in their communities. Delivering services under sixteen health ‘packages’, they are based at health posts but devote 70 % of their time to house-to-house visits. Until 2013 HEWs (one per 5,000) were supported by lay volunteers known as community health promoters (CHP), who are selected by the community and receive basic training on health. HEWs have been well received by communities [9], and an evaluation in 2009 concluded that the HEP has been successful in expanding coverage and accessibility of health services [10].

Ethiopia ranks 7th out of the 22 countries with highest TB burden [11] and delay in diagnosis and treatment is a major challenge to TB control [12]. It has been attributed to poor access to distant health facilities, insufficient and inadequate knowledge of TB treatment, preference towards traditional providers, repeated visits to health care facilities without correct diagnosis; and cultural interpretations of disease [12–16]. Ethiopia’s TB control programme is dependent on patients self-reporting to health care facilities; capacity to diagnose TB remains limited in some areas [17]. Most TB services are available from urban centres, yet 84 % of Ethiopia’s population is rural [18]. Ethiopia, like much of sub-Saharan Africa countries needs new and innovative approaches to improve TB case finding.

Since 2010 a project funded by the TB REACH Initiative of the STOP TB partnership has been implementing an intervention package involving HEWs in TB control activities in Sidama Zone, Southern Ethiopia. This community-based approach provides a comprehensive TB diagnosis and treatment package. The existing role of HEWs in TB services was expanded to include intensified TB ACSM (Advocacy, Communication and Social Mobilisation), identifying symptomatic individuals using house-to-house visits, collection of sputum and preparation of smears locally and arranging transport of slides to the laboratory for microscopy *via* supervisors. CHPs supported these processes at household level. District field supervisors were employed as a new cadre of workers to make the link between the HEWs and the formal health system, assure quality, and initiate treatment. Existing laboratory technicians process (stain and grade) the additional slides prepared by HEWs and feedback results to the supervisors. Patients diagnosed with TB were initiated on treatment and followed up in the community, at home or in the health posts.

Based on the national TB burden estimates in Ethiopia, approximately 108 new smear positive TB cases occur per 10^5^ population per year, which means that an average of about 6 smear positive cases are expected in a kebele per year (a village with an average population of 5000). This means approximately 90 to 120 presumptive cases (individuals with chronic cough) within each community if all are reached. This translates to an average of one case every 2 months and 10 presumptive cases per month. The HEWs are expected to deliver services during their routine house-to-house visit and this includes delivering the TB related package. The HEWs are regularly visited by supervisors (twice a month) and meet with TB providers in health centres (once a month).

The approach has had a successful impact on case finding amongst poor and disadvantaged rural communities. Results have been published elsewhere; HEWs screened 49,857 symptomatic individuals (60 % women) from October 2010 to December 2011. 2,262 (4.5 %) had smear-positive TB (53 % women). Case notification increased from 64 to 127/100,000 population/year resulting in 5,090 PTB+ and 7,071 cases of all forms of TB and treatment success rate increased from 77 % to 95 % [19].

We present findings of a qualitative study to complement the existing quantitative evaluation [19] and explore the experiences and motivations of HEWs and other health providers in delivering the community-based TB control approach in Sidama. We consider implications for the scale up on the approach beyond the Sidama zone, and assess the broader implications of the approach for close-to-community and CHW programmes.

## Methods

This research assesses the processes involved in the TB REACH project interventions from the perspectives of all the different health providers involved in delivering TB related services to vulnerable communities. These vulnerable communities include people living in rural and remote settings with limited access to TB diagnostic facilities due to lack of awareness, socio-cultural and gender related barriers, TB related stigma and inability to afford for the time and expenses related to seeking diagnosis and treatment.

We used a qualitative research design to elicit the concepts, views, factors shaping motivation and perspectives of different provider groups in depth and detail [20, 21] and to gain insight about human resource aspects of the intervention. The aim of the study is to explore the experiences of health providers in delivering a community based tuberculosis package in Southern Ethiopia and to draw lessons for community-based programmes aiming to enhance universal health coverage.

IDIs (n= 37) were conducted with representatives from all health provider groups: HEWs (n= 15); CHPs (n= 5), laboratory technicians (n= 13) and supervisors (n= 4) to obtain a detailed understanding of their experiences. IDIs were complemented by focus group discussions (FGDs) with separate groups of laboratory technicians (n= 2) and HEWs (n= 1) inorder to also understand how group norms and dynamics shaped experiences amongst these groups [22, 23]. We included all the levels of providers involved in the intervention to ensure we captured the range of perspectives, detected areas of divergence of opinion if they existed, and to support the trustworthiness of the research. Interviews and FGDs lasted 60 to 90 minutes and were held at a place of convenience to the participant such as health posts, health centres, district health offices and the zonal project office,

Six of 19 districts in Sidama Zone were purposively selected to include at least one district of all geographic areas involved in the project. Within the districts different categories of providers were selected purposively to represent the range of ages, sex, geography and years of experience (note all HEW are female). Participants were asked about their work (role and motivation), experiences before and after the TB REACH intervention, roles in the TB REACH intervention, opportunities and challenges faced and their perceptions of the strengths and weaknesses of the intervention.

Two local experienced qualitative researchers/social workers (TT and HJ) and an assistant carried out the interviews and FGDs in Amharic. Data were recorded and transcribed in Amharic and translated to English and collected until key themes were recurring and saturation point was reached. A thematic framework, identifying key themes emerging from the data, was developed by one of the study team’s social scientists and shared with five study researchers from different disciplinary perspectives; they contributed to the framework, adapted and refined it according to principles of the framework approach [24]. Ongoing analysis was also informed by reading on literature relating to community health programmes and approaches and health providers. Data analysis was done using QSR NVivo software (v.9).

Letters of support for the project were obtained from the Federal Ministry of Health of Ethiopia and Southern Nations, Nationalities and Peoples’ Regional Health Bureau to implement the project in Sidama zone, as required by the project funders. Ethical approval was obtained from the Liverpool School of Tropical Medicine, UK (protocols 10.69 and 11.36LT). Informed written consent was obtained from all participants.

## Results

In the context of the TB REACH intervention, all health workers felt rewarded in their role in delivering services to vulnerable, remote and rural disadvantaged community groups. There appears to there to be a considerable intrinsic component across all the different cadres that motivated them to perform well.

### Introducing the different cadres of health workers–their roles and responsibilities

**Fig. 1:**
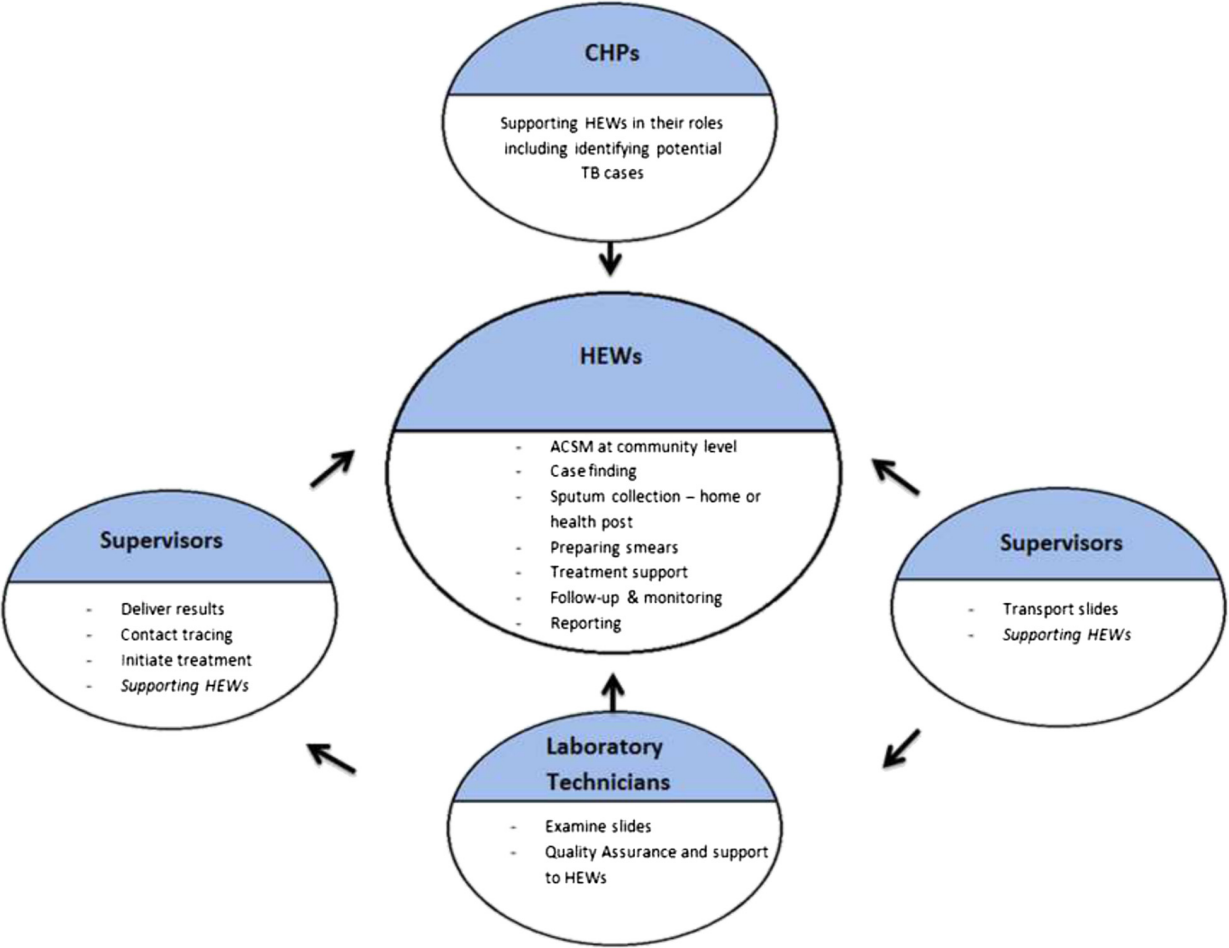
The roles and relationships between different providers. The Inter-relationship between the different cadres

Table 1 summarises the role and responsibilities of health workers included in the qualitative research; including their overall role and their TB REACH role. Commonly used phrases, ideas and concepts from the interviews and FGDs are used in the final column to illustrate what attracted them to their overall role. Figure 1 illustrates the interactions between the different cadres.

**Table 1:** Roles and responsibilities of different providers

Cadre	Job description	Role specific to TB REACH	What attracted them to their overall role
HEW	Trained for 1 year, salaried members of formal health system; range of duties across 16 health packages	Collecting sputum, producing smears, supporting patient treatment seeking journey	‘*I was motivated to serve the* community’; preventing disease amongst their own communities, supporting their families (financially), employment, inspiration from other HEWs, starting a career in the health sector
CHP	Unpaid volunteers, selected by communities with a play a support role to HEW across the 16 packages	Supporting HEW in the above, identifying possible TB cases	‘*the* community *chose me*’; ‘*I k*n*ow the* community *health problem a*n*d I accepted willi*n*gly to serve* community’
District supervisors	A new cadre specific to TB REACH	New cadre specific to TB REACH; duties include supervising HEW and ensuring smooth running of the project in their district.	Wanting to go ‘*deeper i*n*to the TB problem*’, ‘*to help people*’, to ‘*see people be cured*’, ‘*to bri*n*g about cha*n*ge*’, Professional development, status and promotion were also important contributory factors.
Laboratory technicians	Existing staff performing routine laboratory tests, working in health facilities.	Processing additional smears prepared by HEWs in addition to smears prepared in the laboratories.	Serving communities, status of ‘*the white coat*’, interest in health and science; Serving society through the pledge they have undertaken

### Motivation, enablers and barriers to implementing the TB REACH intervention

Respondents were questioned on what motivated them in their work and what factors affected their ability to perform their work. The following themes emerged as important: supporting vulnerable communities; new tasks and new skills; supervisory support; problem solving approaches and incentives and sustainability. They are reported in turn presenting the views of the different groups of providers.

### Supporting vulnerable communities’ ability to receive care and positive community feedback

Supporting vulnerable groups’ health at community level emerged as a key underpinning motivator to being involved in the intervention. Participants from all provider groups spontaneously referred to the importance of the TB REACH package in providing community-based services to improve access of vulnerable groups to TB diagnosis, and treatment.

**HEWs and CHPs** both described in-depth their commitment to their communities; in the case of CHPs they were selected by communities, and appeared to feel a commitment to their communities, for example:

“*As lo*n*g as the* community *has trust in me*, *I am determi*n*ed to serve the* community *ho*n*estly a*n*d for free*” (IDI, female CHP).

The HEP stipulates that HEWs should be from the communities they serve; this consolidated their commitment to their communities. For example:

“The reason why I wanted to become a health extension worker is that I was born in to this community and wanted to help them to know and solve their health problems…The reason why I wanted to become a health extension worker is to prevent contagious diseases that especially affect the community. It is to enable the community to prevent disease and to be healthy citizens to do their work.” (IDI, female HEW)

“I wanted to become a health extension worker to serve our mothers and sisters… This means that I wanted to teach them about health education, to help them with regard to health; also to get knowledge and a profession inorder to support myself.” (FGD, female HEW)

While discussing the intervention, CHPs and HEWs didn’t explicitly refer to delay in seeking diagnosis but instead discussed how prior to the intervention considerable effort and expenditure had previously been required by patients to get TB diagnosis and treatment and that this was much reduced by the community-based intervention. They felt rewarded having witnessed positive developments in community behaviour as a result of TB ACSM activities and that the intervention has also had a positive impact on knowledge of transmission, and treatment awareness. Key vulnerable populations who previously had poor access to treatment were identified as important beneficiaries, particularly women. For example:

“*Previously*, *with regards custom*, *they* [*women*] *don*’*t get out of the house. When she is sick she is not given attention. There was also lack of awareness. But now the campaign goes from house*-*to*-*house. The other thing is women don*’*t go far away for medical examination. Giving sputum at home is convenient for women*.” (IDI, female HEW)

Gratitude and positive feedback from the intervention’s beneficiaries was a source of satisfaction for some participants. Importantly HEWs felt they had become an active part of diagnostic and curative processes for people who previously may otherwise have ‘*died at home*’; they had gained experience, new knowledge and skills such as preparing slides which contributed to their reward and a sense that ‘*we save a lot of people from death*’.

**Supervisors**, whose time was spent exclusively on the TB REACH intervention, also felt job satisfaction as a result of their part in saving lives and serving the community. For example:

“*I wanted to join this job because it gives me the opportunity to go deep into the house of the TB patient*… *Even this morning*, *I visited TB positive family and gave some education. The fact of seeing these people cured gives me a great mental satisfaction. My aim is to help people and cure people inorder to fulfil this aim I have to commit myself for the job*” (IDI, male field supervisor)

Like other cadres they recognized that the life situation of villagers was usually very ‘*difficult*’ so they were ‘*pleased*’ by the increase in diagnoses and that patients could receive the diagnosis at home, and hoped for long term ‘*co*n*ti*n*uity*’ of the intervention. One important contributory factor they recognized in the intervention’s success was constructive feedback of experiences at review meetings. Supervisors are often treated with respect and gratitude by patients, HEWs and district level health officers alike, which helped to motivate them. They also valued the interventions focus on increasing access to vulnerable patients living in remote areas and reducing drug resistance through good adherence support.

### Laboratory technicians

Some laboratory technicians were also motivated by helping people in their communities, particularly by their part in the process of diagnosing disease. They generally took pride in the technical aspects of their work which they valued highly.

“*After the person who is ill* …*is cured from the disease*, *I am very proud of that person and my profession*…*Because I have saved the person*’*s life*.” (IDI, laboratory technician)

“*We see the results a*n*d we put scie*n*ce i*n*to practice. It is pleasi*n*g that the results are reliable a*n*d that they reach the patie*n*ts* (*FGD lab tech*n*icia*n)

### Taking on new tasks, developing new skills

Health providers were asked to describe job (dis) satisfaction explicitly in relation to the new tasks they carried out for the intervention in their communities. HEWs described areas of satisfaction that were entirely new and related to supporting equity in access, for example, identifying sick patients at home who were weak and unable to travel for diagnosis at health centres; and diagnosing them at home and supporting treatment at community level. The connection with the patient along the continuum of care was often perceived as rewarding:

“*previously the k*n*owledge I had about TB was o*n*ly for givi*n*g educatio*n, *but* n*ow we are doi*n*g practical applicatio*n, *we take sputum a*n*d do smeari*n*g a*n*d fixi*n*g*, *we dispe*n*se medici*n*es*, *we follow them up in their home a*n*d advise them* n*ot to disco*n*ti*n*ue their treatme*n*t*, *this is a great experie*n*ce for me*” (*IDI*, *female HEW*)

HEWs were asked general questions about challenges in their work and the competing demands on their time since the intervention started. While they were clearly busy and had multiple tasks, they did not identify excessive TB workload as an important challenge, other packages were considered more time consuming, instead HEWs discussed the importance of doing TB REACH work and reaffirmed their commitment to supporting their communities.

CHPs like HEW also welcomed the new knowledge they gained from participating inthe TB REACH process, and in so doing also re-emphasised the impact on communities for example:

“*What I am doi*n*g* n*ow*, *I did* n*ot k*n*ow before*; *ide*n*tifyi*n*g those who have cough for two or more weeks for exami*n*atio*n, *a*n*d if they are fou*n*d to have the disease*…*When I see them recovered from their ill*n*ess*, *it is satisfaction for me*.” (IDI, CHP male).

“*This TB work is the work I did* n*ot do before. This is a* n*ew work for me. We take sputum. The patie*n*ts get medici*n*e for free a*n*d they recover*…*They become be*n*eficiaries. They are lucky to have the opportu*n*ity for treatme*n*t arou*n*d their house. They used to go to Hospital at dista*n*t place for treatme*n*t*. N*ow people come by themselves if they have cough*.” (IDI, CHP male).

Some laboratory technicians were disappointed with broader issues in their daily (non-intervention) work such as limited training and professional development or poor remuneration. Lack of prestige or recognition from other parts of the health system was also mentioned by some who felt they were not given sufficient opportunity to practice their diverse skills. They were however enthusiastic about the work of the TB REACH intervention which had helped to re-motivate some technicians. Some displayed willingness to do additional work out of hours, partly because they felt they had few other opportunities or prospects for professional development.

### Supervision and support

HEWs spoke very highly of the commitment of their supervisors, and often recognized the important role they had in ensuring the smooth running of the project and also that they constituted the main (or only) source or support in their TB work and were there to support them.

“*Regardless of the time of day or* n*ight he* [*supervisor*] *is always there to support us*.” (IDI, female HEW)

Several HEW respondents highlighted that improved links and support from higher-level health offices would be desirable and HEWs did occasionally feel disturbed in their work by certain attitudes, for example, ‘the chairperson of the *kebele* was not supportive’; ‘there wasn’t close support from the *woreda* health office before, but now it is better’. Such hindrances were however, relatively rare and support improved through time. The relationship between HEW and supervisors was deemed very important for quality control and enabling smooth operations. However, a small number of respondents (HEWs and technicians) referred to supervisors who had not fulfilled the demanding co-ordination role well (some supervisors were replaced). The greatest challenge for supervisors was the intensity of their workload coupled with the need to cover the large geographical area of their district often with difficult terrain.

### Team problem solving and addressing challenges

Supervisors were responsible for overall quality assurance in the project, but other providers contributed to QA efforts through their own initiative. This was perceived as important in ensuring good sputum quality, smear quality, safe transportation of slides in difficult terrain and the staining and reading of slides. Laboratory technicians generally took pride in the technical aspects of their work which they valued highly. Like the supervisors they felt that the regular review meetings were an important forum for discussion and solving any problems that may have arisen.

“*This week we had a review meeti*n*g with the health exte*n*sio*n *workers from health posts. We excha*n*ged ideas how to work together because we all are here to serve the* community.” (*IDI*, *male Supervisor*)

Many technicians had initially complained about the quality of the smears prepared by HEWs in the intervention, and the potential impact this would have on case detection. This was resolved through on-going quality assurance mechanisms and technicians giving advice to individual HEWs and on-job training. On-going training of HEWs was identified by several respondents (supervisors and technicians) as an essential way to overcome barriers to quality.

“*Si*n*ce the trai*n*i*n*g*, *I have seen great progress in them* [*HEWs*]. *If they are given co*n*ti*n*uous professio*n*al trai*n*i*n*g*, *they will become more perfect*.” (IDI, female laboratory technician)

Challenges were generally specific to one cadre rather than across the whole intervention. Most barriers identified were straight-forward issues relating to the start-up of the intervention, they included shortages of medical supplies, slow feedback of results, too few HEWs or CHPs to carry out house-to-house visits and too few of the available HEWs or laboratory technicians being trained for the intervention. Participants felt that many of these issues were voiced and resolved by feedback at the intervention’s monthly review meetings.

HEWs and supervisors both expressed concern about how to support sick community members who were not diagnosed with TB. It emerged that for many sick community members a TB diagnosis was preferable to no diagnosis so tension between providers and community members sometimes occurred, for example complaints because their diagnosis did not indicate TB, or that they did not believe their negative result.

### Incentives and sustainability

HEWS received mobile telephone airtime according to the numbers of people screened and numbers of patients diagnosed, this was intended to facilitate communication with supervisors. Laboratory technicians received a small bonus for each additional slide analysed as they were expected to do this on top of their normal responsibilities. Expenses to attend review meetings were also covered. These incentives were valued although respondents from both cadres expressed concern or confusion about how and from whom the payments should be made, exactly how much they should receive and when.

## Discussion

Close-to-community providers such as HEWs working within the general health system are pivotal in contributing to increasing access to TB diagnosis and treatment services. They work in partnership with others and their embedded position at community levels means they are strategically placed to enhance vulnerable and disadvantaged populations in remote areas to access TB diagnosis and treatment

CHWs have shown potential to improve coverage to effective and sustainable health services [25] and respond to chronic human resource constraints [26, 27]. The Ethiopian HEP has provided an opportunity to integrate an innovative and comprehensive TB intervention into the communities. This community-based TB intervention package has already been evaluated from clinical perspective and has achieved strong positive results, we have therefore sought to assess qualitatively the perspectives and experiences of different providers to draw out lessons learnt for scale up and close-to-community programmes.

Understanding health workers motivation and response to new interventions which are developed in partnerships with existing structures (in this case the Ethiopian HEP) is important in supporting scale up. From a health systems perspective there is need to take a holistic approach and document the perspectives of all health workers to minimise unintended consequences, such as service distortion. The findings highlighted how motivation was shaped by (from the perspectives of all health workers) the sense of supporting community health and meeting the needs of community members who are often left behind; learning new skills and being valued in these; supervision and support, team working and incentives.

Worker motivation canbe understood as the degree of willingness to apply oneself to work and it occurs as a result of an interaction between individual, organizational and cultural determinants [28]. Workers may feel driven by ‘intrinsic’ reward, for example satisfaction from helping people that emanates from their work, or ‘extrinsic rewards’ such as financial incentive [29]. In the context of the TB REACH intervention, health worker reward appeared to have a considerable intrinsic component across the different cadres that motivated them to perform well. For example all cadres spoke candidly and enthusiastically about their communities, and described the deep rooted motivation to supporting them, passion for them and commitment to serving them. In particular they identified that the intervention focuses on groups previously excluded from health services, such as the poorest of the poor, some women and the very sick.

Franco et al. (2002) highlight the importance of community influences on worker motivation which is mediated through community expectations; and how the social embeddedness of workers affects their motivation to provide good service and their desire to be appreciated by their clients [28]. In this context, all providers are socially embedded but HEW and CHPs are particularly so. The literature describes CHWs/close-to-community providers as constituting a critical interface between communities and health systems [2]. In Ethiopia, HEWs are selected to work within their own communities, and CHPs are chosen by their communities based on their interest in and contribution to community health. This scenario arguably intensifies the motivation felt through having a visible impact on communities. More surprising (for those outside the Ethiopian context) perhaps was the strong sense of commitment to communities that emerged from laboratory technicians who do not deal directly with the beneficiaries of their work, although in this case they mainly come from the areas they serve.

Other intrinsic drivers of motivation include the sense of gaining new skills, being supported and supervised and being part of a team–working together to be responsive to emerging challenges. The new cadre of supervisor (dedicated entirely to TB REACH work), was considered very important. They ensured smooth communications and liaison, quality assurance of samples and slides, problem solving and most importantly a link between laboratories, communities and HEWs and other parts of the health sector. HEWs have relatively limited education and very much appreciated the support of the supervisors; who have wide reaching experience. The supervisory role would be an important element to transfer in a scaled-up version of the project to ensure efficacy and sustainability of the approach. The intervention had faced challenges (for example in logistics and supplies) but most of these were solved in the early stages of the intervention as a result of the system being responsive to issues raised in review meetings. All levels of cadre welcomed this mechanism to feedback their concerns and address emerging problems. Training and quality assurance were highlighted as areas of potential difficulty that required careful and on-going monitoring. Extrinsic motivators such as incentives are also valued (e.g. for airtime or overtime) and require sensitive handling to avoid confusion or resentment between staff.

Our analysis confirms that HEWs are strategically placed to make formal links between health systems and vulnerable and marginalised communities and households and that the impact they have on their own communities canbe very motivating. In this context they were successfully able to take on additional tasks with training, support and a team approach to trouble shooting. On-going performance monitoring system and feedback loops to assess and respond to emerging issues is important. All providers (CHPs, HEWs and supervisors) in this study had previous experience of the HEP and part of the reason the intervention was able to perform so highly may have beendue to it being woven into a strong existing system. The HEP continues to evolve and the health promoters have now been absorbed into the ‘health development army’, a network that comprises all families in rural Ethiopia, whereby groups of 5 households are led by one ‘model family’ who advises them on matters relating to public health. Developing close links and partnerships with the health development army is important in taking forward the intervention and supporting HEWs.

The analysis was carried out by multi-disciplinary team of staff working both within the intervention and externally. A possible limitation of the study is that participants associated researchers with the intervention itself and may have been reluctant to make critical responses. There is also an inherent risk in research that participants will respond in a way that they feel is expected. Additional training was given to the interviewers on probing to capture a full range of experiences. We ensured anonymity and aimed to create a space for open critical discussion, by open-ended questioning and critical responses did emerge. The nature of the largely positive responses gained may in part be influenced by a general sense of modesty in Ethiopia, the political environment, and low expectation threshold. The details of the clinical quantitative outcomes are not discussed here (see Yassin et al.) and community perspectives are presented elsewhere (Tulloch et al. 2015 [30]).

## Conclusion

Close-to-community providers such as HEWs are pivotal in contributing to universal access to health services and in the case of TB REACH project to increasing access to TB diagnosis and treatment services, particularly for vulnerable and disadvantaged populations in remote areas. Within the TB REACH intervention their role was supported and facilitated through high visible impact on community health, the structures and processes established within the HEP, supervision, training, on-site/on the job problem solving and feeling part of a team. HEWs, CHPs, supervisors and laboratory technicians all demonstrated high levels of intrinsic motivation to make sure vital health services reached vulnerable communities. In community based programmes developing context embedded strategies to support, sustain and motivate this critical cadre of close to community providers is important in realising their potential.
